# How Aortic Stiffness in Postmenopausal Women Is Related to Common Cardiovascular Risk Factors

**DOI:** 10.1155/2014/216080

**Published:** 2014-07-16

**Authors:** Maria Maiello, Annapaola Zito, Marco Matteo Ciccone, Pasquale Palmiero

**Affiliations:** ^1^ASL BRINDISI, Cardiology Equipe, District of Brindisi, Via Francia 47, 72100 Brindisi, Italy; ^2^Cardiovascular Diseases Section, Department of Emergency and Organ Transplantation (DETO), University of Bari, Italy

## Abstract

*Objective.* Our study investigates major common cardiovascular risk factors relation with aortic stiffness on 269 postmenopausal women by global pulse wave velocity (PWVg), useful to relate PWVg to risk of major cardiovascular events. *Patients and Methods.* Women were categorized as hypertensive (H), hypercholesterolemic (C), or diabetic (D). Aortic stiffness was assessed by PWVg measured with pulsed Doppler, at the left ventricular outflow tract (LVOT) and at the right common femoral artery. *Results.* All population mean PWVg was 8.2 m/s. 85 (26.5%) women were H; mean PWVg was 7.9 m/s. HC women were 118 (36.7%), with mean PWVg 8.3 m/s. HD women were 30 (9.5%), with mean PWVg 7.8 m/s. HDC women were 36 (11.2%), with mean PWVg 9.3 m/s. 52 (16.1%) menstruate women without risk factor were control group (CG), with mean PWVg 6.5 m/s. Highly significant was the statistical difference in PWVg between HDC women and each other group: *P* < 0.0005 versus CG; *P* < 0.01 versus H; *P* < 0.03 versus HC, and *P* < 0.05 versus HD. No difference in PWG was observed comparing the other groups. There was difference for age among all groups, except for CG, made by younger women. *Conclusion.* PWVg was highly increased in postmenopausal women affected by hypertension, diabetes, and hypercholesterolemia all at once. Hypertension is the major determinant for PWVg. The only addition of diabetes or hypercholesterolemia did not increase significantly PWVg. Our study supports the usefulness of the assessment of aortic stiffness as a marker of cardiovascular disease.

## 1. Background

Quantitative assessment of arterial structure and function by noninvasive methods on populations is essential for a better understanding of the physiopathology of the vascular diseases. Noninvasive measurement of pulse wave velocity is an easy, safe, and reproducible method for assessing the aortic arterial stiffness [1]. It is well known that both the ageing process and elevated blood pressure are associated with alterations in vascular structure and function [2–4].

These alterations include widening and wall thickening of large arteries and decrease in central arterial compliance (arterial stiffness) [2]. Several decades ago, it was suggested that aortic compliance measurements might be used as an indicator of atherosclerosis [5, 6]. However, despite numerous investigations concerning the role of risk factors on the structure and function of large arteries, the relationships between arterial wall hypertrophy for atherosclerosis and arterial stiffness have been investigated by specific population studies [7, 8].

Recently, increased arterial stiffness, assessed by an increased pulse wave velocity (PWV), was demonstrated to be a risk factor for atherosclerotic cardiovascular disease [9–12].

Arterial stiffness is known to increase with aging. Furthermore, it is unclear how the possible relationships between different cardiovascular risk factors, particularly age and hypertension, can improve the atherosclerosis process determining arterial stiffening.

The goal of our study was to investigate major determinants among common cardiovascular risk factors for aortic stiffness on 321 consecutive women using an echocardiographic method to calculate global pulse wave velocity (PWVg), [13, 14] useful to relate the PWVg to the absolute risk of major cardiovascular events through cardiovascular risk factors estimation.

## 2. Methods

### 2.1. Subjects

We screened 269 consecutive postmenopausal women by our heart station who referred to us by their general practitioners for baseline evaluation. We included in our study women on menopause only after, at least, twelve consecutive months of amenorrhea. All of these postmenopausal women were either natural or surgical, while 52 menstruate women without any cardiovascular risk factor were our control group (CG). The mean age of the patients was 59.9 ± 12 years. Individuals were categorized as hypertensive or not, according to ESC 2013 Guidelines [15]. Hypercholesterolemia was assessed according to 2013 ACC/AHA guideline on the assessment of cardiovascular risk [16]. Diabetes mellitus was diagnosed according to 2013 Standards of Medical Care in Diabetes by American Diabetes Association [17].

Exclusion criteria were systolic heart failure assessed by diagnosis of LV ejection fraction <50%, wall motion abnormalities, coronary artery diseases, severe valvular diseases, atrial fibrillation on enrolment, pulmonary hypertension estimated from tricuspid regurgitation velocity by the modified Bernoulli equation, renal failure assessed by serum creatinine >1.2 mg/dL, and major noncardiovascular diseases as cancer or chronic lower respiratory tract disease.

### 2.2. Materials and Methods

**Figure 1:**
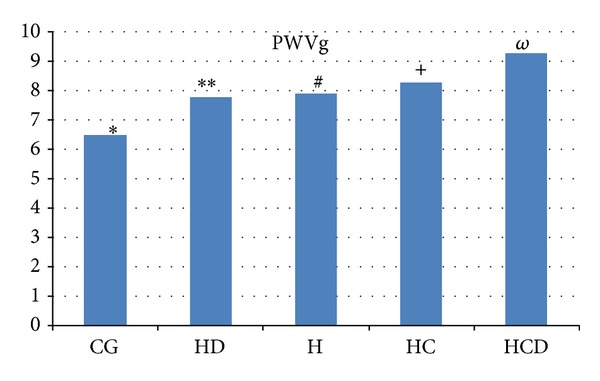
Significant statistical difference in PWVg between HDC women and each other group. *ω* versus *P < 0.0005, *ω* versus **P < 0.05, *ω* versus ^#^ *P* < 0.01, and *ω* versus ^+^ *P* < 0.03. CG: control group, H: hypertensive postmenopausal women, HD: hypertensive and diabetic postmenopausal women, HC: hypertensive and hypercholesterolemic postmenopausal women, HDC: hypertensive, diabetic, and hypercholesterolemic postmenopausal women, PWVg: pulse wave velocity global.

Aortic stiffness was assessed by PWVg measured in a partially supine position with the head of the examining table elevated by 30° after resting for at least 10 minutes. The examination was carried out with pulsed Doppler (3.5 MHz probe) using two-dimensional (2D) guidance and ECG trigger GE Vivid 3 Expert, HealthCare, via Galeno 126, Miland (Italy), was used which is an echo-Doppler system equipped with a multifrequency transducer. The interval between the beginning of the QRS complex and the foot of the systolic upstroke in the Doppler spectral envelope was calculated (Figure 1) and averaged over five consecutive cycles, nonsimultaneously (but at the same heart rate) at the LV outflow tract site and at the right common femoral artery. The PWVg was calculated between the LV outflow tract and right common femoral artery by dividing the straight line distance between suprasternal notch and right common femoral artery by the transit time. The distance was assessed using a tape measure located at the same place as the ultrasound probe. The transit time was defined as the difference between two intervals of time using the Doppler method. This method has just been validated about reproducibility by two previous studies [13, 14]. However, recordings were separated by only a few seconds for a given patient and this method was applicable in all subjects. The Doppler images were recorded on hard disks to be analyzed later using the calipers of the echo machine. In all of the patients, intraobserver variation in PWVg was studied and interobserver reproducibility was analyzed independently by two trained ultrasonographers.

### 2.3. Laboratory Measurements

The plasma total cholesterol, high-density lipoprotein cholesterol, triglycerides, and plasma glucose levels of the subjects were measured enzymatically. All the blood samples were collected in the morning after an overnight fast. The plasma low-density lipoprotein level was calculated using Friedewald's formula [18].

### 2.4. Statistics

Data on PWVgs were available for all women. The general characteristics of the study population were described by the mean ± standard deviation (SD). The association between PWVg measurements and cardiovascular risk factors was evaluated using a Student's *t*-test for continuous variables and Chi-square test for categorical variables. A *P* value <0.05 was considered significant.

## 3. Results

All population mean PWVg was 8.2 m/s. Eighty-five (26.5%) women were hypertensive (H), mean age was 59.7 years, and mean PWVg was 7.9 m/s. Hypertensive and hypercholesterolemic (HC) women were 118 (36.7%), mean age was 63.5 years, and mean PWVg was 8.3 m/s. Hypertensive and diabetic (HD) women were 30 (9.5%), mean age was 61.8 years, and mean PWVg was 7.8 m/s. Hypertensive, diabetic, and hypercholesterolemic (HDC) women were 36 (11.2%), mean age was 61.9 years, and mean PWVg was 9.3 m/s. Fifty-two women without any cardiovascular risk factor were our control group (CG), mean age was 49.7 years, and mean PWVg was 6.5 m/s (Table 1). There was a highly significant statistical difference in PWVg between HDC women and each other group: *P* < 0.0005 versus CG, *P* < 0.01 versus H, *P* < 0.03 versus HC, and *P* < 0.05 versus HD (Figure 1). No significant statistical difference in PWVg was observed comparing the other groups. There was no significant statistical difference for age among all groups, except for CG, made by younger women.

## 4. Discussion

The main finding of our study is a statistical significant relationship between PWVg increase and a cluster of cardiovascular risk factors as hypertension, diabetes, and hypercholesterolemia in a population of postmenopausal women. Arterial stiffness is thought to be a prognostic marker in patients with atherosclerotic cardiovascular diseases [8, 10–12]. Our results demonstrated that there was no significant statistical difference for age among all groups, except for CG, made by younger women. While large systemic studies have demonstrated an association between age and arterial stiffness, these studies did not investigate the prevalence of the menopause and also did not evaluate the association between age and arterial stiffness in a narrow age range (such as the early postmenopausal period) [19, 20]. Several previous studies have examined the association between arterial stiffness and the menopause. London et al. demonstrated, based on a cross-sectional study, that the menopause improves the age-related increase in arterial stiffness; however, the number of subjects in their study was too small [21]. Otherwise, Smulyan et al. reported, again based on a cross-sectional study, that structural changes of the blood vessels, rather than the menopause, influenced age-related changes in PWV in women; however, they did not specifically consider the influence of the menopause in their study [22]. Thus, the association between the menopause and arterial stiffness remains unclear. Our previous study demonstrated that postmenopausal women have an increased aortic stiffness compared with controls when assessed using PWVg. Hypertensive status has an additive effect on aortic stiffness in postmenopausal women, but its role is independent of menopausal status [13]. When assessing the effect of the menopause on arterial stiffness, the influence of age is difficult to avoid. Nevertheless, estrogen deficiency in perimenopausal women has been reported to progress gradually until nearly 60 years of age [23]. The menopause is known to have an unfavorable influence on conventional atherosclerotic risk factors, like the serum cholesterol level, blood pressure, and body weight [24, 25], thereby accelerating the progression of atherosclerosis and influencing arterial stiffness. Several studies have reported that hypercholesterolemia and diabetes mellitus are associated with increased arterial stiffness. Lebrun et al. demonstrated a significant association between PWV and body mass index, fasting blood glucose, and serum triglyceride levels in postmenopausal women; however, they failed to evaluate the corresponding associations in premenopausal women. Therefore, they could not definitively conclude whether the aforementioned associations were specifically menopause-related [26]. The prevalence of hypertension, diabetes mellitus, hypercholesterolemia, and obesity was significantly higher among the postmenopausal women; a significant relationship between the menopause and the brachial-ankle PWV was observed, independent of age and conventional atherosclerotic risk factors [27]. Therefore, estrogen deficiency may, at least in part, augment the age-related increase in arterial stiffness during the early postmenopausal phase. In addition, estrogen deficiency activates the renin-angiotensin system, induces the production of atherogenic inflammatory cytokines, and reduces collagenase activity [28–30]. While these factors may contribute to the menopause-related augmentation of arterial stiffness, the precise factors influencing the correlation between the menopause and arterial stiffness could not be determined in the present study. Furthermore, the increase in brachial-ankle PWV according to age was larger in this study than that for PWV measurements performed using conventional methods [19, 31]. Brachial-ankle PWV is strongly correlated with aortic PWV. Some studies have suggested that brachial-ankle PWV can be used as a marker of atherosclerotic vascular disease [32, 33]. However, brachial-ankle PWV measurements include not only central arterial stiffness but also peripheral arterial stiffness. Age-related changes in the peripheral component (especially for arterial stiffness in the lower extremities) might contribute to the larger increase in brachial-ankle PWV according to age seen in the present study, at least partially. Therefore, further evaluations are needed to examine the applicability of brachial-ankle PWV as a marker of cardiovascular risk. Carotid femoral PWV is a marker of cardiovascular risk [34] and it is calculated from the data recorded at the level of the common carotid and femoral arteries but in fact represents more the PWV between the descending thoracic aorta (at approximately 10 cm of the aortic arch) and the common femoral artery patient. For these reasons we have adopted PWVg; it is calculated between the LV outflow tract and the right common femoral artery; the measurement is performed at the same heart rate, which represents global PWV (PWVg) that better estimates global aortic stiffness. Unfortunately, this method is an option useful only for physicians with an echocardiographic and Doppler machine at their disposal.

## 5. Conclusion

PWVg is highly increased in postmenopausal women affected by hypertension, diabetes, and hypercholesterolemia all at once. As just assessed in our previous paper, and confirmed here, hypertension is the major determinant for PWVg on postmenopausal women. Only the addition of diabetes or hypercholesterolemia did not increase PWVg in a significant way, while the addition of both did it. Our study supports the usefulness of the assessment of aortic stiffness as a marker of cardiovascular disease and to identify at an early stage subjects at risk of cardiovascular events.

## 6. Summary Table

- Noninvasive measurement of pulse wave velocity is an easy, safe, and reproducible method for assessing the aortic arterial stiffness.
- Increased arterial stiffness, assessed by an increased pulse wave velocity (PWV), was demonstrated to be a risk factor for atherosclerotic cardiovascular disease.
- Arterial stiffness is known to increase with aging; it is unclear how the possible relationships between different cardiovascular risk factors, particularly age and hypertension, can improve the atherosclerosis process determining arterial stiffening in postmenopausal women.
- PWVg is highly increased in women affected by hypertension, diabetes, and hypercholesterolemia all at once.
- Hypertension is the major determinant for PWVg on postmenopausal women. Only the addition of diabetes or hypercholesterolemia did not increase PWVg in a significant way, while the addition of both did it.
- Our study supports the usefulness of the assessment of aortic stiffness as a marker of cardiovascular disease.

## Figures and Tables

**Table 1:** PWVg in the different postmenopausal women groups and control group.

Women	Group	*N*	Et$\overset{`}{\text{a}}$	PWVg (m/sec)
All population		321	59,9	8,2
Hypertensive	H	85 (25%)	59,7	7,9
Hypertensive hypercholesterolemic	HC	118 (36,7%)	63,5	8,3
Hypertensive diabetic	HD	30 (9,5%)	61,8	7,8
Hypertensive diabetic hypercholesterolemic	HDC	36 (11,2%)	61,9	9,3
Control group	CG	52 (16,1%)	49,7	61,5
